# Giant Isthmocele With a Large, Degenerated, False Broad Ligament Fibroid and Its Diagnostic Dilemmas: The Use of the Halloween Sign

**DOI:** 10.7759/cureus.51055

**Published:** 2023-12-25

**Authors:** Tamilselvi Sethupathy, Madhankumar Madathupalayam, Krithika Arun Prasad

**Affiliations:** 1 Obstetrics and Gynaecology, CK Medical Center Hospital, Erode, IND; 2 Minimally Invasive Surgery, CK Medical Center Hospital, Erode, IND; 3 Anesthesiology and Perioperative Medicine, CK Medical Center Hospital, Erode, IND

**Keywords:** isthmocele, hysterolaparoscopy, halloween sign, mri, false broad ligament fibroid, large isthmocele

## Abstract

The prevalence of isthmocele or caesarean scar niche is increasing nowadays. This is largely attributed to the increasing rate of caesarean deliveries globally, although the growing awareness among gynaecologists and assisted reproductive technology specialists may contribute to the more frequent diagnosis of isthmocele. Although the presenting complaints, diagnosis, and management of isthmocele are discussed extensively in the literature, this case of large isthmocele with a large degenerated false broad ligament myoma, which caused diagnostic dilemma both preoperatively and postoperatively, needs a special mention. This is one such case of large isthmocele rarely reported in perimenopausal women obscured by concomitant uterine pathology like degenerating large broad ligament fibroid. This also stresses the need for awareness and application of various techniques, such as the Halloween sign, for the proper diagnosis of the emerging but treatable complication of the most common surgery performed worldwide.

## Introduction

Although fibroids are the most common tumours of the reproductive age group, common symptoms like menorrhagia, pelvic pain, and dysmenorrhoea almost masquerade the isthmocele [1]. Among the various locations of fibroids, broad ligament fibroids are uncommon. An isthmocele is a common cause of secondary infertility [2]. Our patient is in the perimenopausal age group and presented with similar complaints almost 22 years after two previous caesarean sections. The associated degenerated false broad ligament fibroid presented a confounding factor in the preoperative and intraoperative diagnosis. There is a paucity of literature showing concomitant reports of such a large isthmocele with false broad ligament fibroid, especially the diagnostic difficulties of the treating clinician and the operating laparoscopic surgeon.

## Case presentation

This 46-year-old patient, who had two caesarean sections, with the last childbirth about 22 years ago, presented with complaints of severe dysmenorrhoea and menorrhagia with intermenstrual bleeding for the past 10 months. Her cycles were 8-12/28 days, although they were irregular earlier. She had a screening ultrasonogram done four years ago by the author, which showed a posterior wall subserous fibroid measuring 4 x 5 cm with no evidence of degeneration. Her ovaries were normal. The patient was asymptomatic then. The ultrasonogram, done both transabdominally and transvaginally, revealed the uterus measuring 7.3 x 4.6 x 5.4 cm, and a long tubular cystic structure was imaged anterior to the uterus, measuring 7.3 x 3.4 cm and adherent to the mixed echogenic mass measuring 9.2 x 8.4 cm. The right ovary was imaged separately. The left ovary could not be imaged separately (Video 1).

**Video 1:** Transabdominal ultrasound showing heterogenous mass with tubular cystic structure imaged anterior to the uterus.

Various differential diagnoses, such as large left-ovarian malignant mass, degenerated subserous fibroid, hydrosalpinx with subserous fibroid, and fibroid with malignant transformation, were entertained. Never was the diagnosis of isthmocele thought of. The patient was advised to undergo computed tomography. The report gave a differential diagnosis of (1) malignant ovarian mass and (2) subserous fibroid with degeneration. As there was a concern about malignant ovarian mass, CA 125 was done, the value being 23 u/ml (0-35 u/ml). The diagnostic dilemma and further plan of either staging laparotomy or laparoscopic surgery could not be solved with computed tomography. An MRI was advised. It revealed a uterus measuring 7.3 x 4.5 x 4.7 cm. The ovaries appeared normal. A large isthmocele measuring 7.3 x 3.4 cm was seen anterior to the uterus. A large degenerated fibroid measuring 12.8 x 8.4 cm was seen in the right lateral wall. Hence, a diagnosis of a large isthmocele with a large degenerating subserous myoma was established (Video 2).

**Video 2:** MRI with contrast showing a large isthmocele and degenerated fibroid in the right lateral wall of the uterus.

Our patient was extensively counselled and then planned for hysterolaparoscopy. A hysteroscopy was done. The uterine cavity was obscured by bleeding (Video 3).

**Video 3:** Hysteroscopy showing bloody discharge continuously draining from the uterus, obscuring the view.

A 10 mm port was introduced in the modified Palmer's point. Another two working ports were introduced in the left iliac fossa and suprapubic region. The uterus was bulky. Both the ovaries and fallopian tubes appeared normal. A large cystic mass, about 12 x 12 cm, was seen in the right lateral uterine wall. The round ligament was stretched over the boggy mass. There was difficulty in identifying the isthmocele and degenerated fibroid as they were adherent to each other and within the two leaves of the broad ligament.

Various techniques were tried. The introduction of the Hegar dilator, which helps in the identification of the site of the defect, could not aid in the diagnosis, as the isthmocele was from the right lateral edge of the scar. The landmark of using peritoneal lateral fields could not be used in such a large isthmocele. Then, with the simultaneous use of a hysteroscopic light source and a laparoscopic camera with the light dimmed, the Halloween sign finally helped in the identification of the isthmocele (Video 4).

**Video 4:** Halloween sign.

Then, as the broad ligament was opened, the isthmocele was seen extending from the right lateral edge of the previous caesarean scar to the lateral pelvic wall laterally and cranially to the degenerated false broad ligament fibroid. The isthmocele was incised, and altered blood was seen escaping through (Video 5).

**Video 5:** Laparoscopic video of a giant isthmocele and broad ligament fibroid.

Since our patient had completed her family, and owing to the large size of the isthmocele and the degenerated broad ligament fibroid, a laparoscopic-assisted hysterectomy was done. In cases where repair of the isthmocele is necessitated for future pregnancies, we definitely need exact identification of the isthmocele, which may be obscured by concomitant uterine pathologies. Such a large isthmocele requires multiple modalities of diagnosis, both preoperatively and postoperatively, and perhaps extensive management options and difficulties too. The Halloween sign elicited by using both a laparoscopic light source and a hysteroscopic light source, where the light is thrown by a hysteroscope and the laparoscopic light source is dimmed, clinched the exact extent of isthmocele in our case.

## Discussion

Prevalence

Although the prevalence of isthmocele is generally reported at 19.4-88%, it is increasing at an exponential rate due to increasing caesarean deliveries throughout the world, improved methods of diagnosis and management of isthmocele due to advances in hysterolaparoscopic techniques, and the eagerness to bear children in advanced age [3]. There are no exact reports of isthmocele with ovarian mass or coexistent large fibroids, as in our case, though there are reports of coexistent endometriosis and isthmocele seen in the literature. Among extrauterine leiomyomas, broad ligament fibroids are rare by themselves and known to cause diagnostic confusion with ovarian malignancy.

Aetiology

According to Vervoort et al., among various aetiologies of isthmocele, the first is a low incision in the well-effaced cervix and mucous secretion that dilates the sutured rims of the myometrium and impairs healing [4]. In our case, though the first surgery was done in labour, the exact details of the stage of labour are not known. The difficulty in identifying the lower uterine layer in the effaced cervix in caesarean sections performed in the second stage of labour may contribute to the faulty closure. This may be overcome by taking stay sutures during a caesarean before making the hysterotomy incision. The second hypothesis is related to surgical technique, where an improper closure of the deeper muscular layer leaves the endometrial layer, thus causing the development of isthmocele [4]. But could the use of catgut, as was the practice in those days, and the use of locking sutures for achieving haemostasis in suturing the hysterotomy wound be the cause, is an unanswered question. According to Hosseini et al., the prevalence of isthmocele was higher in the catgut group. The residual myometrial thickness was greater in the Vicryl group (4.98 cm ± 2.18) compared to the catgut group (3.70 cm ± 1.50) [5].

The third hypothesis proposes early adhesion between the caesarean scar and the anterior abdominal wall, pulling the edges of the wound and leading to isthmocele. This becomes exaggerated in a retroflexed uterus, in which those counteracting forces are increased [4]. In our patient, this hypothesis seems to be the plausible cause, as the pulling force of the degenerating enlarging fibroid, which was adherent to the surface of the previous caesarean scar, led to the formation of such a large isthmocele as the fibroid enlarged. Though the author performed an ultrasound five years ago, probably the small size of the isthmocele or the lack of awareness of this complication could be the reason for non-identification.

Although the timeline for the isthmocele formation from the time of the caesarean section finds no mention in the literature, the last childbirth being more than 22 years in our patient is equally intriguing. There are various trials in which an ultrasound is scheduled after six months of caesarean section to identify the isthmocele. According to van der Voet et al., the uterine scar after a caesarean section is not a static feature and changes over time. The residual myometrial thickness between the niche and the bladder at the site of the caesarean section scar measured with sonohysterography decreases significantly over time between two and 12 months after a caesarean section [6]. Does all isthmocele need to be identified as causing alarming anxiety to the patient, and does all cause symptoms, is still an enigma.

Diagnostic methods

Transvaginal ultrasonography and saline infusion sonography are commonly available methods for detecting isthmocele. According to Roberge et al., the proportions of suspected scar defects detected by hysterography, sonohysterography, and transvaginal ultrasound were 58%, 59%, and 37%, respectively [7].

Marotta et al. suggested that evaluation of uterine scar defects after caesarean section can be done using ultrasound and MRI, and the laparoscopic repair gives good postoperative anatomic and functional outcomes [8]. Since the size of the defect was large and appeared cystic on transabdominal ultrasound, the presence of a large degenerated myoma obscured further additional information in transvaginal ultrasound. Although hysterography could have been performed, the persistent bleeding from the uterine cavity and the differential diagnosis of isthmocele were never thought of, so it was not done. Hence, a large isthmocele can be clearly identified by MRI, as in our case. According to Zawin et al., MRI is superior to ultrasound in the evaluation of the entire pelvis in women with leiomyomas [9].

Fiaschetti et al. reported a case of large isthmocele on the anterior wall of a retroflexed uterus misdiagnosed as a uterine cavity filled with menstrual blood during a previous hysteroscopy, where an MRI gave a definitive diagnosis, as in our case [10]. MRI is the preferred method for accurately defining pelvic masses and the site of origin because it is sensitive in identifying uterine fibroids and it can readily demonstrate the uterine zonal anatomy, which helps in establishing the communication between the isthmocele and the lower uterine cavity. Leiomyosarcomas are large heterogeneous masses due to haemorrhage, confounding the degenerative changes in the large fibroid.

According to Dueholm et al., transvaginal ultrasonography is as efficient as MRI in detecting myoma presence, but its capacity for exact myoma mapping is limited, especially in large (>375 mL) and multiple myoma (>4) [11]. According to Panayotidis et al., up to 5% of patients reported to have uterine fibroids by CT were subsequently found to have ovarian tumours at the time of surgery [12]. MRI provides better discrimination of the source of the pelvic mass [13]. Typical leiomyomas demonstrate low to intermediate signal intensity on T1-weighted images and low signal intensity on T2-weighted images. Myxoid degeneration and necrosis may be visible as high-signal-intensity areas on T2-weighted images. Another common variant seen on both T1- and T2-weighted images is a cobblestone-like appearance due to hyaline degeneration, with high-signal-intensity foci representing areas of infarction due to rapid growth [14]. This additional information from the MRI clinched the diagnosis of both co-existing pathologies.

Fiocchi et al. reported that 3T-magnetic resonance diffusion tensor imaging was better than transvaginal ultrasound for evaluating the thickness of the scar [15]. According to Wang et al., MRI is usually used for a preoperative work-up, and uterine contrast-enhanced MRI is a much better imaging method to measure the thickness of residual myometrium and the length, width, and depth of the caesarean scar defect than a general MRI [2]. Since our patient had coexistent pathologies, the use of transvaginal ultrasound or hysterography may not yield a definitive diagnosis, but an MRI is a diagnostic modality.

Size of the isthmocele

The size of the isthmocele is determined based on residual myometrium. The size of the isthmocele here could be classified as a large defect, as the residual thickness measured is negligible, although various studies excluded coexistent pathologies. Isthmocele is classified as large if the size of the defect is >50% of the residual myometrial thickness.

Shape

The shapes of the isthmocele are triangular or semicircular when limited laterally, though round, oval, and droplet shapes and inclusion cysts are also described. The most common shapes were semicircular (50.4%), triangular (31.6%), droplet-shaped (10.3%), and inclusion cysts [6]. In our case, it shows an oblong bulge towards the peritoneal cavity, probably directed by the pulling force of the large fibroid.

Intraoperative techniques

During the surgery, differentiating the isthmocele was challenging. Various techniques are used by surgeons to identify the isthmocele intraoperatively. After mobilizing the bladder from the lower uterine segment, a Foley catheter was inserted into the uterine cavity through the cervical canal and the balloon of the catheter was filled, allowing clear identification of the isthmocele pouch under laparoscopy [16]. By hysteroscopy, the isthmocele is identified by the diverticular mucosal hyperplasia, and then the hysteroscopic light is directed towards the cephalic limit of the scar defect. Laparoscopic lights are decreased in intensity, and the "Halloween sign" is identified by hysteroscopic transillumination. The caesarean scar was easily identified by inserting a uterine probe through the cervix into the dehiscent scar [17]. The isthmocele was identified with intraoperative transrectal ultrasonography and is particularly useful when a bulge in the caesarean scar is not identified laparoscopically [18]. The identification of the defect using intraoperative transvaginal ultrasound was useful when the repair of the isthmocele was attempted in pregnancy to simultaneously visualize the foetal cardiac activity [19]. A Hegar dilator has been used during the successful laparoscopic repair of an isthmocele [20]. The use of lateral bands, a consistent anatomical landmark first reported by Dr. Sandesh Kade of India, which identifies the level and width of the uterine niche, is another intraoperative diagnostic technique [21].

Awareness of the various intraoperative techniques could aid the operating surgeon in clearly defining the extent of isthmocele, which would otherwise result in recurrence, frustrating both the patient and the clinician.

Manag​​​​ement

An exact diagnosis of isthmocele is essential to avoid treatment like oral contraceptives and antibiotics, and extensive investigations like CT and tumour markers. The various modalities of treatment are hysterectomy and laparoscopic-assisted vaginal hysterectomy, as done in our case, and this decision was made owing to the large volume of the uterus and the degenerated fibroid. Excision of the scar tissue and repair of the myometrium by vaginal route, hysteroscopy, laparoscopy, or V-notes are other methods tried successfully. Though there is emerging consensus regarding management based on the residual myometrial thickness overlying the isthmocele, the cutoff for hysteroscopic repair being 3 mm, and other cases successfully repaired by the laparoscopic method, the final rule is not out. Our patient has given consent for a hysterectomy in view of our counselling of the very large size of the isthmocele, and she had no plan of fertility preservation either.

## Conclusions

In the new era of increasing caesarean sections, this case of a very large isthmocele stresses the need to consider isthmocele in the differential diagnosis of pelvic masses, as it mimics various other pathologies in perimenopausal women. The technical expertise of the operating surgeon and the associated uterine pathologies, like the large fibroid in our case, should be the decision-making factors for a successful management strategy. Further, the crucial factor is the intraoperative identification of the isthmocele with awareness and anticipation of the technical difficulties and necessary set-up in a low-resource setting that would otherwise hamper the correction of the recurring complication even if proper repair is done in a woman determined to preserve her fertility.

Isthmocele is an emerging complication of the most common surgery performed all over the world. Large isthmocele can masquerade as many other concomitant uterine and adnexal pathologies. MRI can have an edge over other diagnostic modalities in such pathologies. Various intraoperative techniques, like the Halloween sign, need to be tried to properly identify the exact extent of the isthmocele.
